# A scalable exemplar-based method for aligning biological taxonomies

**DOI:** 10.3897/BDJ.14.e191754

**Published:** 2026-06-17

**Authors:** Jonathan Rees, Nico M Franz, Beckett Sterner

**Affiliations:** 1 Arizona State University, Tempe, United States of America Arizona State University Tempe United States of America; 2 University of Kansas, Lawrence, United States of America University of Kansas Lawrence United States of America

**Keywords:** Catalogue of Life, ChecklistBank, Mammal Diversity Database, region connection calculus, species checklist, taxonomy alignment, taxonomic concept

## Abstract

This article describes an exemplar-based approach to species checklist alignment. Biologists in many fields often work with tables whose rows denote taxonomic groups (more formally called taxonomic concepts). Such tables, here called checklists, are essential ingredients for communicating the information contained in many research articles, databases developed for research projects and laboratories and comprehensive taxonomic resources, such as the Mammal Diversity Database and Catalogue of Life. An important activity is reconciling or *aligning* two or more checklists (also called taxonomic concept mapping), i.e. relating the records of one checklist to the records of another. Our implementation of exemplar-based alignments is scalable both pragmatically and computationally: it does not require labour-intensive manual processing of the checklists or alignments, it handles large checklists efficiently and it is usefully aware of taxonomic concept relationship mappings. Open-source code implementing the exemplar-based approach is available online as part of the List Tools software library.

## Introduction

Biologists in many fields often work with tables whose rows denote taxonomic groups (more formally called taxonomic concepts) (Franz and Peet 2009, Ingenloff et al. 2025). Such tables, here called checklists, are essential ingredients for communicating the information contained in many research articles, databases developed for research projects and laboratories and comprehensive taxonomic resources, such as the Mammal Diversity Database (MDD) (Burgin et al. 2018, Burgin et al. 2025) and Catalogue of Life (COL) (Bánki 2022). Recognising the growing number and importance of checklists, the COL and Global Biodiversity Information Facility (GBIF) (Slota and Bowker 2015, Franz and Sterner 2018, Heberling et al. 2021) have recently deployed a new public data resource called ChecklistBank (Döring et al. 2022, Bánki et al. 2023, Hernández-Robles et al. 2023). An important activity is reconciling or *aligning* two or more checklists, i.e. relating the records of one checklist to the records of another (Cheng and Xia 2023). In database terms, we would like to join the two tables with awareness of semantics. Whenever one works with multiple checklists, alignment is an important first step in data analysis, data integration and quality control (Grenié et al. 2022). As each checklist may have thousands of taxonomic groups, scalable solutions to checklist alignment are highly desirable.

This article describes an exemplar-based approach to checklist alignment. Exemplars are sample members from the groups to be aligned. Our implementation of exemplar-based alignment is scalable both pragmatically and computationally: it does not require labour-intensive manual processing of the checklists or alignments, it handles large checklists efficiently and it is usefully aware of taxonomic concept differences. Typical current practice is to match checklist records based on names and nomenclatural relationships (such as synonymy), either naïvely or with allowance for differences in scientific names to reduce false negatives (Boyle et al. 2013). This may be followed by manual curation. The set of matches is then used as an alignment. There are tools, such as PhyloMatcher (Rader et al. 2023), the Taxonomic Name Resolution Service (Boyle et al. 2013), gnparser (Mozzherin et al. 2017) and taxamatch (Rees 2014), to facilitate name-based alignment. However, a name-based match does not imply congruent taxonomic concepts: a single name could refer to larger, smaller or otherwise overlapping groups or two names could refer to the same group (Remsen 2016, Franz et al. 2016a). False negatives and positives can lead to errors when reasoning about information of biological importance (Sterner and Franz 2017, Franz and Sterner 2018, Pyle et al. 2022, Finkbeiner et al. 2025). One is faced with the prospect of either tolerating the mismatches or the laborious process of finding and fixing them. While it is possible to use a general logical inference engine to obtain more precise results, such methods require very well curated inputs and they run slowly and with problem-size limitations (Chen et al. 2014, Franz et al. 2016b). For additional related work, see (Hruschka et al. 2024) and (Cheng and Dinh 2025) .

Two lines of research combine here. The method of 'split' comparison (Redelings and Holder 2017), which is essentially the same as exemplar set comparison as described below, arises out of work on phylogenetic tree synthesis and conflict analysis. The use of automated theorem proving in taxonomic checklist alignment (Franz and Peet 2009) is motivated by the problems of integrating and resolving the incomplete and heterogeneous taxon information that taxonomists and taxonomy users confront. The current work represents a bridge between these two.

List Tools is a free software package for checklist alignment and related list operations, such as converting to Darwin Core (Wieczorek et al. 2012), cleaning, sorting, subsetting and exemplar extraction. We illustrate the results List Tools generates by applying it to align the Mammal Diversity Database (MDD) v.2.0 checklist with the mammals section of the 2024 annual Catalogue of Life checklist. Our discussion is meant to introduce the idea and utility of exemplars for taxonomic concept alignment to a broad audience without assuming deep technical familiarity with theories of biological classification or the inner workings of biodiversity data infrastructures.

The method we present is useful for multiple applications, including comparisons of checklists from different sources or versions from the same source at different times. For example, taxonomic authorities could use List Tools to generate documentation of differences (“diffs”) in taxonomic concepts between versions. Organisations managing lists of threatened or protected species may find List Tools useful to accelerate the labour-intensive process of aligning multiple input taxonomies to a locally managed checklist. Reports from List Tools should help in locating and transferring information from one taxonomy into a second that is being brought up to date. The reports produced by List Tools also contain useful information for validating the completeness and accuracy of nomenclatural information, for example, for systematists working on a taxonomic revision.

For readers already familiar with taxonomic alignment methods, we briefly highlight the availability of a robust set of exemplars as a key assumption of the method and we also explain this in more detail below. One source of exemplars is the type specimens implied by the scientific names present in the checklist records. This is the method currently provided by the software. However, exemplars are not limited to type specimens as long as they can be accurately placed in groups in both checklists. Other sources of evidence, such as geographic ranges, digital images, genetic sequences and morphological traits, might provide the basis for generating exemplars (Upham et al. 2022, Finkbeiner et al. 2025). The exemplar-based approach, therefore, has the potential to be further expanded in scope and generality in future work.

In the remainder of the paper, we first introduce the ideas of taxonomic concepts and alignments. Then we describe some background assumptions and resources that are useful to describe the relationships between taxonomic concepts, including the Region Connection Calculus-5 (RCC-5) (Thau et al. 2008, Thau et al. 2009) and the semantic hierarchy assumed by the Darwin Core data standard. Next, we present the List Tools alignment method and an application aligning two mammalian taxonomies. We close with discussing future work.

## Taxonomic concepts and alignments

List Tools consists of a number of checklist manipulation tools, but here we are concerned foremost with the method implemented by its exemplar-based alignment tool. While we put intuitions and pragmatics in the foreground, we will sometimes, for clarity, describe the method using conventional first-order logic terminology of formulae (formal sentences that might be true or false), models (relative to which every formula is either true or false) and axioms or assumptions (formulas or formula schemas true in all models). We start by introducing the main ideas and assumptions needed to understand the alignment method.

### Taxonomic concepts

We use the word *occurrence* generically for individual living (or formerly living) things, whether named or not or collected or not. Each record in a checklist is associated with a grouping of occurrences. We call such a grouping a taxonomic concept (Franz and Peet 2009) and we call the occurrences the taxonomic concept's members. Occurrences are not treated explicitly in checklists, but rather are biological entities summarised in bulk by a checklist’s records.

Biologists rely on preserved evidence, commonly known as physical or digital vouchers, to document particular occurrences. A voucher may be an individual organism, preserved specimen, tissue sample, photograph or other material serving the same purpose. Vouchers also play a role in documenting taxonomic concepts. In modern taxonomic practice, a distinguished occurrence called the type specimen, possessing its own voucher(s), is given for each named concept. Additional occurrences might also be specified for use in a taxonomic concept description.

A checklist record's taxonomic concept is described in prose somewhere in the scientific literature and a human specialist can usually locate the description starting from the name and other information in the record, through potentially laborious literature and database search. Studying descriptions is one way to determine taxonomic concepts and how they relate to one another. However, descriptions are rarely cited unambiguously in checklists and even when they are, they are not in a form that can be used computationally. Despite immense progress making published descriptions available in digital, machine-readable formats (Cui et al. 2016, Penev et al. 2019, Folk et al. 2024, Zhang et al. 2025), they are, at present, typically suitable only for non-automated activities when comparing checklists.

### Comparing taxonomic concepts using RCC-5

There are many ways to compare records and many ways to compare taxonomic concepts. We focus our effort on Region Connection Calculus-5 (RCC-5) relationships, which let us express information about taxonomic concepts that is concise, biologically meaningful and important (Franz and Peet 2009, Franz and Thau 2010). For two sets x and y, the possible RCC-5 relationships are as follows:

- congruence (x and y have the same members), written x = y
- inclusion (every member of x is a member of y), written x < y
- subsumption (x contains every member of y), written x > y
- disjointness (no occurrence is in both x and r), written x ! y
- non-trivial overlap (none of the above: x and y have members in common, but x and y are different and neither contains the other), written x >< y

Exactly one of these five relationships holds between any two sets, in particular between any two taxonomic concepts.

We sometimes write x <= y, y >= x and x != y with the obvious meanings (“includes or is congruent to”, “subsumes or is congruent to” and “is not equal to”, respectively). This approach is compatible with the Taxon Concept Schema 2 standard ratified in December, 2025 (Taxon Concept Standard Maintenance Group 2025). We say x and y *intersect* if their relationship is other than ! and are *comparable* if their relationship is =, < or >.

The heart of the alignment functions of the List Tools is a utility for comparing taxonomic concepts (specified by their records) and estimating the RCC-5 relationship that holds between them.

### Interpretation of Darwin Core

The List Tools alignment method takes, as input, two checklists. A checklist is a strict hierarchy of accepted names or taxa decorated with synonyms (meant in the technical sense of biological nomenclature). The choice of checklist representation is somewhat arbitrary and we use Darwin Core because it is in widespread use for checklist exchange. List Tools also includes optional preprocessing tools for data cleaning and converting alternative hierarchy formats to Darwin Core. To reason formally using Darwin Core checklists, we have to commit to a way to interpret Darwin Core and warn users that the tool might be unsuitable if those assumptions are not met in their particular checklists.

Some definitions for present purposes:

1. A record is an *accepted record* if its taxonomicStatus column contains “accepted” or “valid”;
2. A record is a *synonym record* otherwise;
3. The *parent record* of a record r is the record, if any, whose primary key (taxonID) is in r’s parentNameUsage column;
4. The *accepted record* of a record r is the record, if any, whose primary key is in r’s acceptedNameUsage column.

Following general usage in taxonomy, whoever prepared the checklist is suggesting that the accepted names (those belonging to accepted records) are or should be in community use and synonyms (those belonging to synonym records) are being replaced, or are to be replaced, by their accepted names.

A Darwin Core checklist is a sequence of rows, each of which corresponds to some taxonomic concept. In some circumstances involving synonyms or "monotypic" parent-child relationships, the method does not make any commitment as to whether the records correspond to distinct taxonomic concepts or the same taxonomic concept.

As a well-formed constraint, the parent of an accepted record, when there is one, is assumed to be accepted, because this is nearly always the case and exceptions would make inference considerably more complex. Similarly, the accepted record of a synonym is assumed to be accepted.

Each record, whether accepted or a synonym, is assumed to have an associated taxonomic concept, even if it is difficult to know what that taxonomic concept is. We might say the associated taxonomic concept is whatever was intended by the checklist preparer, who presumably acts according to the taxonomic community’s norms and strives to be understood.

From our experience with Darwin Core files, it is usually safe to constrain “parent” and “accepted” axiomatically as follows. Suppose that r and s are records and tc(r), tc(s) their associated taxonomic concepts. We use the RCC-5 vocabulary above to express relationships amongst concepts. Then:

1. If r is an accepted record and s is r’s parent record, then tc(r) < tc(s);
2. If r is a synonym record and s is r’s accepted record, then tc(r) <= tc(s);
3. If (a) r and s are distinct records, (b) r and s are both accepted and (c) r and s have the same parent record, then tc(r) ! tc(s) (i.e. accepted siblings are disjoint).

These axioms simply express that the accepted records form a single-inheritance hierarchy, with overlap allowed between synonyms’ taxonomic concepts and those of their siblings. The axioms are complete for accepted records. Overall, they are incomplete: for example, the relationship between two synonym records with the same accepted record could be any RCC-5 relationship (the intended relationship can sometimes be constrained using heuristics such as name matching).

Sometimes, particular Darwin Core files violate these assumptions. For example, there can be two synonym records that are pretty clearly intended to designate the same taxonomic concept (perhaps same genus, epithet and authorship), but they have distinct parent records. For some use cases, these problems can be tolerated, but it is best to correct or remove such records before the checklist is processed.

Other fields used in processing:

- taxonID, providing a unique key for every record;
- scientificName, which for a species would ordinarily be a binomial combined with an authority string, for example, “Ornithorhynchus anatinus (G. K. Shaw, 1799)”. This is subject to data cleaning and parsing;
- taxonRank. The method for the most part is insensitive to rank, but occasionally rank helps in making fine discriminations.

Fields not mentioned above are ignored.

### Exemplars

On their own, checklists cannot tell us much about the large, overwhelming universe of occurrences in the natural world. In particular, we do not have enumerations of the members of most taxonomic concepts. This method relies on a sampling of the full occurrence universe to obtain a smaller, tractable model universe for the checklists being aligned, one in which proxies for taxonomic concepts can be enumerated.

Each sample occurrence, to be useful for comparisons, must have a known classification in both checklists. That is, we have to know which taxonomic concept it falls under in each checklist. Any occurrence that is only known in one checklist is not used as a sample.

Let's call these samples *exemplars*. They exemplify the taxonomic concepts that contain them. Each record r has an 'exemplar set' es(r) consisting of all the exemplars contained in the record's associated taxonomic concept. In a taxonomic concept hierarchy, an exemplar is contained in all ancestors of the record.

A set of exemplars is required as an input to the alignment process. As exemplar sets can be written to and read from files, the option is open to use any source of exemplars, such as photographs, genetic sequences or physical specimen records. If a user of the software has not prepared an exemplar set through other means (genetic etc.), exemplars are computed from the checklists as follows.

While checklists do not normally give direct information about occurrences, we do know that each record — at least at species level and lower — has an associated type specimen belonging to the record’s associated taxonomic concept, by the information in the record (especially a ‘name’ similar to the type specimen's true protonym) and the rules of taxonomic nomenclature (Pyle 2004). If two records in the two checklists are known to have the same associated type specimen, the shared type specimen can be used as an exemplar. The taxonomic concepts might be different, but at least they have that exemplar in common.

We can also learn about the taxonomic concepts intended for a checklist by how it groups records as synonyms to accepted names. Synonyms via their type specimens leave behind a trace of taxonomic events that occurred in the past. By “events”, we mean taxonomic changes proposed in the published literature and taken up by later taxonomists, such as the ones who prepared the checklists. For example, a judgement that a set of occurrences forms two species instead of one is a common event and it leaves behind a deprecated species name as a synonym. If two checklists reflect taxonomies prepared before and after this change, the remaining species name’s record in the later checklist will likely be associated with more exemplars than the earlier checklist’s record for the same name, reflecting distinct taxonomic concepts for the two.

When an objective synonymy holds according to nomenclatural history and records r and s are records for the taxa involved, the corresponding concepts will have a type specimen in common, which can therefore be an exemplar. A subjective synonymy likely implies tc(r) incongruent with tc(s) and might or might not share any occurrence. It is useful when a checklist calls out objective synonyms using the taxonomicStatus field when epithets differ. Objective synonyms might also be detected via name matching.

### Taxonomic alignment

The output of the method is a taxonomic alignment. A taxonomic alignment consists of a model (complete hypothesis) regarding the RCC-5 relationships that hold between the taxonomic concepts associated with the records of two checklists, consistent with the RCC-5 relationships that hold within the checklists. Each relationship in the alignment is between a taxonomic concept associated with a record in one checklist and a taxonomic concept associated with a record in the other checklist. There may be other alignments consistent with the checklists; the choice is based on what we think are reasonable heuristics. It is best to think of an alignment as an oracle that answers queries, not a data structure listing all true relationships, since such a list would be enormous and unwieldy.

## Material and methods

The method takes two checklists and an optional exemplar set as inputs and yields an alignment consisting of RCC-5 relationships as output.

Checklists are provided as tables, some of whose columns are Darwin Core fields. The method makes use of the fields mentioned above (section “Darwin Core semantics”) and ignores the rest. For each row, there is, by intent, some (unrecorded) taxonomic concept. The taxonomic concepts are not directly defined by the checklists, but only constrained by them.

The exemplars are provided in a table with columns for unique identifier, checklist 1 record key and checklist 2 record key, identifying records for two taxonomic concepts that contain the exemplar.

The problem is to determine, for each pair of records drawn from the two checklists, the likely RCC-5 relationship that holds between the taxonomic concepts associated with those records, according to heuristics given below. The relationship is proposed by considering membership of exemplars in the taxonomic concepts. Internally, the software realises an alignment procedurally and if disjointness relationships (the vast majority) are omitted, the alignment can be written out as a table. Taxonomic concepts intersect if and only if they are not disjoint (they share at least one occurrence). If two records are associated with intersecting taxonomic concepts, then any occurrence in their intersection can serve as an exemplar and the absence of such an exemplar from the exemplar set is taken as evidence of disjointness.

Essentially, the method is to deduce what the taxonomic concept relationships would be if they were the same as the relationships between the respective exemplar sets.

### Exemplar collection

If no exemplar set is supplied, we harvest type specimens for all records that occur in the two checklists, both accepted records and synonyms and determine type specimens to use as exemplars by looking for record pairs that match by names, accounting for spelling variations of the sort found in taxonomy. Matching ‘homotypic’ records (those indicating a common type specimen/protonym) is similar to name matching in other taxonomic software for ‘taxon’ matching, requiring similar treatment of spelling mistakes, author and year mistakes, gender changes and genus changes. It differs in permitting any match that implies the same type specimen, such as subspecies name to species name.

We use gnparser (Mozzherin et al. 2017) to parse, stem and normalise names and common heuristics to handle genus changes and various kinds of errors and spelling variation. For efficient processing, we employ a standard technique in database processing called block nested join (Kim 1980), grouping records by epithet, to reduce the complexity of best-match computation and ambiguity detection. The outcome is a collection of equivalence classes of records under a "same type specimen" or "same protonym" relation. The equivalence classes that contain at least one record in each checklist define exemplars.

### Relationship estimation

First, each record is assigned an exemplar set. Exemplar sets are computed bottom up in the obvious way, starting with the assignments of exemplars to ‘tip’ records (usually species) and forming unions of exemplar sets of children and synonyms going towards the root of the hierarchy. We can then compute RCC-5 relationships that hold between exemplar sets. If both exemplar sets are non-empty, call this relationship an ‘estimate’ because it is suggestive of the true relationship between the records’ intended taxonomic concepts. The goal is to find the RCC-5 relationship between the taxonomic concepts for the corresponding records. Table 1 gives possible taxonomic concept relationships as a function of the exemplar set based estimate, showing the effects of potential unsampled occurrences.

Note that tc(r) >< tc(s) is logically consistent with any relationship between exemplar sets. When selecting a relationship to be proposed by a checklist alignment, we prefer any other consistent relationship to tc(s) >< tc(r), since overlap detection would require additional exemplars and is both unlikely and usually unsupported in practice (Vaidya et al. 2018).

When es(r) = es(s), most of the time there are no other records with an exemplar set equal to this one, so it is plausible to suppose that tc(r) = tc(s). Otherwise, we are in the position of inferring relationships without the benefit of exemplar set distinctions. Any solution can be underdetermined, stymied for lack of information or defeated by unusual checklist data. We have chosen to finesse the problem using the rank designations in the Darwin Core records. We assume that, for each checklist, all the records with this exemplar are totally ordered by taxonomic concept containment (<), since otherwise we would have overlap relationships. The two ordered sequences need to be interleaved consistently. If every record has been given a rank (taxonRank), a reasonable heuristic is to use rank comparison for taxonomic concept comparison. For example, if r has rank ‘class’ and s has rank ‘order’, then we propose tc(r) > tc(s). Certainly other approaches are possible.

If either record r or s has no exemplars, we find ancestor records r’ of r and s’ of s, furthest from the roots (“smallest”), that do. We then take the relationships between r and r’, r’ and s’ and s’ and s and compose them (Ragni and Wölfl 2005). When doing so would require composing < with >, the result is undetermined.

### Software Implementation

**Figure 1.**
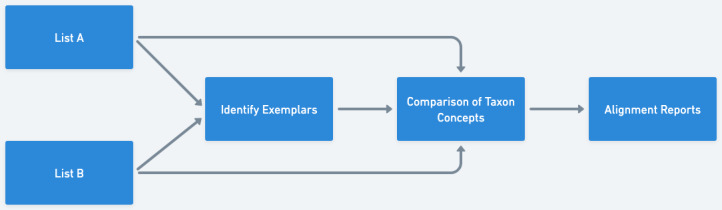
A simple flow diagram showing the key steps in the List Tools alignment method:

A summary flow diagram is provided in Fig. 1. The software has three main jobs:

- Wrangling inputs (checklists, exemplar sets) and outputs (reports);
- Selecting type specimen-based exemplars, if needed;
- Computing estimated taxonomic concept relationships.

Checklists come in Darwin Core format. A conversion script is required when starting with a different format, as is the case with MDD and NCBI Taxonomy. ChecklistBank provides a useful and growing source of Darwin Core formatted checklist files.

Having ingested the two checklists from input files, a complete set of exemplars is loaded (if already available) or computed from the type specimens implied by the checklists. In principle, it is possible to intervene manually or automatically at this point to repair false relationships in the final alignment by repairing or enhancing the exemplar set file, although this is not currently a feature in List Tools.

With these in place, it is readily possible to compute relationships as needed. The totality of these relationships constitutes an alignment of the two checklists. Typically, most of the relationships are disjointness (i.e. es(r) ! es(s)), so that, if disjointness is considered a default for unstated relationships, then we can save considerable space when we turn an alignment into an enumerated data structure or report.

## Results

To illustrate the types of information produced by List Tools, we aligned the recently published MDD v.2.0 (Burgin et al. 2025) to the mammal species listed in the 2024 annual version of COL. The MDD checklist contained 6,759 accepted species records and 34,439 rows total, with the additional rows describing the higher taxonomic backbone and synonyms. The COL checklist contained 6,082 accepted species and 25,664 rows in total, representing the higher taxonomic backbone, synonyms and subspecies. The alignment report has one row for each RCC-5 relationship (other than disjoint) between accepted species and one row for each record with no known relationship to the opposite checklist.

Executing the alignment procedure, including type specimen based exemplar search, took under 30 seconds on a personal Linux laptop computer (3500 MHz max, 16G RAM) and generated 6,129 inferred RCC-5 relationships between the intersecting taxonomic concepts for accepted species-ranked records from both checklists. There were 13,649 exemplars. The main outputs are provided as Table 2 and Suppl. materials 1, 2. Table 2 summarises differences between the two checklists in order of frequency. Most common are the 5,175 RCC-5 congruence (=) relationship between species records accepted in both checklists. Next are a total of 1,187 records that are either present in the COL checklist, but have no known species relationship to MDD 2.0 or present in MDD v.2.0 and have no known species relationship to COL. These represent an aggregate of novel species recognised in the taxonomy literature, names missing from the lists and potential nomenclatural errors that could not be addressed by the data cleaning procedures used by List Tools (c.f. Franz et al. (2016b)). The other RCC-5 categories besides congruence and disjointness (i.e. <, >, ><) account for 954 of the remaining relationships.

### Example 1: A straightforward case of congruence

**Figure 2.**
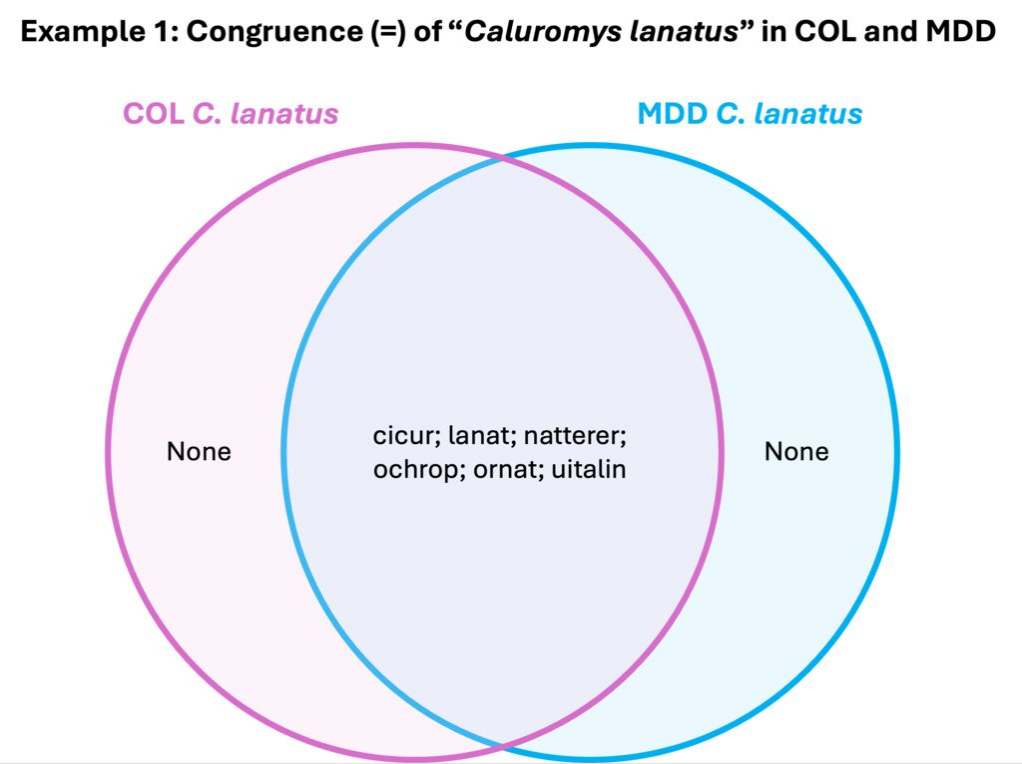
Venn diagram illustrating estimated congruence relationship between concepts for “*Caluromys lanatus*” accepted names in COL and MDD checklists. Exemplars are shown only using their stemmed epithets for brevity. The overlap region corresponds to the Table 2 column of exemplars found in both COL and MDD concepts. Regions labelled “None” do not have exemplars.

In the two checklists, the taxonomic concepts for the name “*Caluromys lanatus*” are estimated to be congruent in meaning. That is, both names are proposed to refer to the same set of organisms, because the associated records in each checklist have the same set of exemplars (see Fig. 2). Exemplar IDs are constructed by combining a number assigned by List Tools with the stemmed epithet of the associated name. For example, the stemmed epithet of “*Caluromys lanatus*” is “lanat” and the associated exemplar is “1091 lanat” in Table 3 under “Exemplars in both A and B concepts”. Other exemplars shown in that column are inferred from synonyms.

### Example 2: A taxonomic "split"

Taxonomists commonly refer to “splitting” a species when they subdivide an existing accepted species into two or more parts. In the case of the deermouse species *Peromyscus maniculatus* (J. A. Wagner, 1845), new evidence of genetic divergence amongst geographic populations led biologists to propose splitting it into up to six species (Bradley and Lindsey 2019, Greenbaum et al. 2019, Boria and Blois 2023). MDD v.2.0 recognises four of the proposed species: *P. sonoriensis*, *P. gambelii*, *P. labecula* and *P. maniculatus* with a reduced circumscription (Mammal Diversity Database 2024). Based on the available online documentation, the corresponding concepts in Catalogue of Life have not been updated since the proposed split. The COL webpage for *P. maniculatus* references the Integrated Taxonomic Information System (ITIS) as its source and reports 30 April 2015 as the latest date of taxonomic review (The Integrated Taxonomic Information System 2025, Catalogue of Life 2025a). The split has important implications for biodiversity data aggregators such as GBIF, which reports 316,249 occurrence records under the name *Peromyscus maniculatus* (Wagner, 1845) as of 30 Dec 2025 (GBIF 2025). The split also has biological significance for understanding zoonotic diseases, since *P. maniculatus* is often claimed to be the primary reservoir host for Sin Nombre virus (*Orthohantavirus sinnombreensis)*. After the split, the range of *P. maniculatus* is restricted to the eastern United States, while *P. sonoriensis* is present in the “four corners” region where the major outbreak of Hantavirus Pulmonary Syndrome in humans occurred in the 1990s (Finkbeiner et al. 2025).

**Figure 3.**
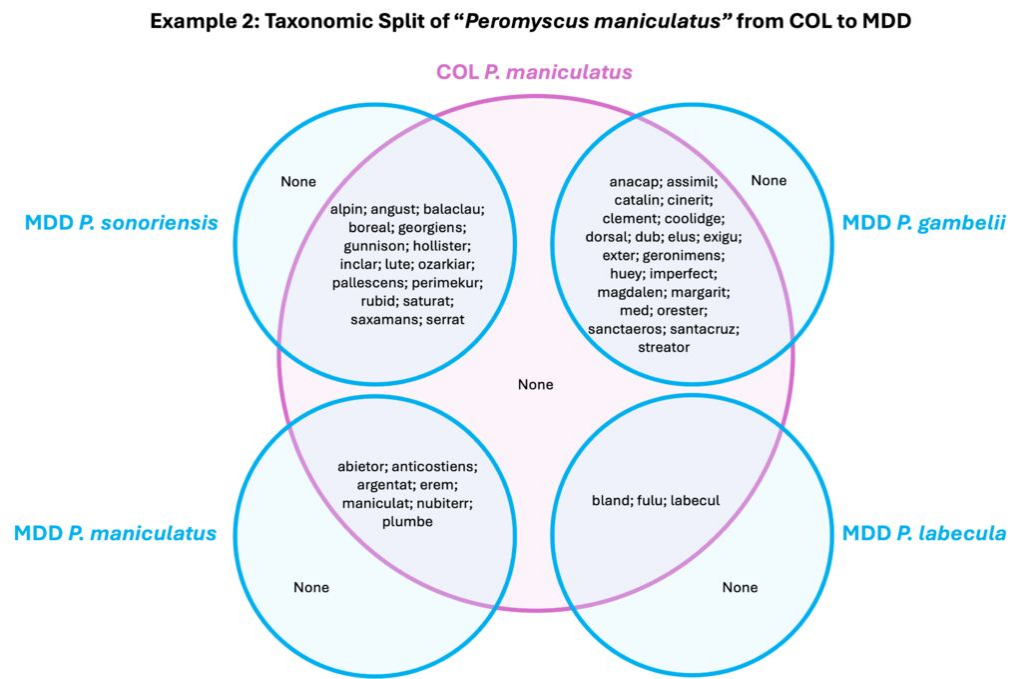
Venn diagram illustrating estimated containment relationships between concepts for “*Peromyscus maniculatus*” in COL and four accepted species names in MDD. Exemplars are shown only using their stemmed epithets for brevity. The overlap regions between pink and blue circles correspond to the Table 3 column of exemplars found in both COL and MDD concepts. Regions labelled “None” do not have exemplars.

Table 4 shows how a sufficient set of exemplars can be harvested from accepted names and synonyms to estimate the 4-way split recognised by MDD since the last update to *P. maniculatus* in COL. This is due in part to the taxonomic complexity and historical interest of the deermice as a group, so that many names have been coined over time and collated as synonyms under both checklists. Fig. 3 visualises how all the exemplars associated with *P. maniculatus* according to COL are divided up amongst accepted names in MDD.

### Example 3: A revision of Microtus species leads to overlap relationship

Another important rodent reservoir host is *Microtus arvalis*, the common vole, which has been linked to multiple viruses such as Tula virus (*Orthohantavirus tulaense*). Biologists have proposed a complex taxonomic revision involving both a split and some reshuffling of synonyms (Tougard et al. 2013, Mahmoudi et al. 2014, Mammal Diversity Database 2025). The corresponding entry for *M. arvalis* in COL shows the same sourcing to ITIS and last update in 2015 as for *P. maniculatus* (Catalogue of Life 2025b).

**Figure 4.**
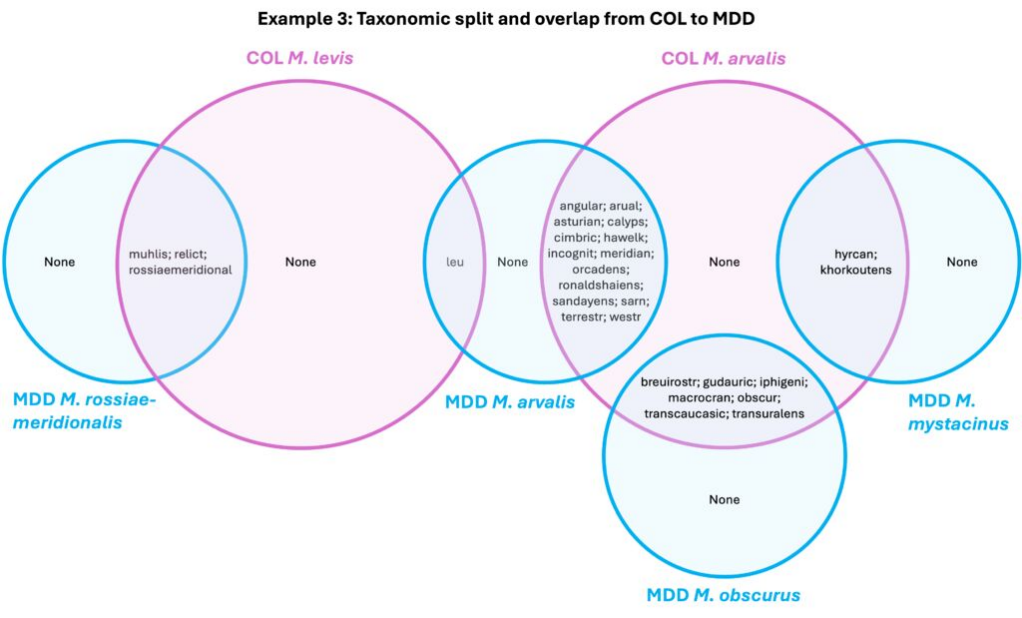
Venn diagram illustrating estimated containment and overlap relationships between concepts for “*Microtus arvalis*” and “*Microtus levis*” in COL and four accepted species names in MDD. Exemplars are shown only using their stemmed epithets due to limited space. The overlap regions between pink and blue circles correspond to the Table 4 column of exemplars found in both COL and MDD concepts. Regions labelled “None” do not have exemplars.

Table 5 shows three estimated containment relationships and one overlap relationship. The accepted species name *Microtus levis* in COL is downgraded to a synonym of *M. arvalis* in MDD v.2.0. The synonyms associated with *M. levis* in COL are instead located under *M. rossiaemeridionalis* according to MDD. At the same time, the names that COL assigns to *M. arvalis* are divided up across three accepted names in MDD: *M. arvalis*, *M. obscurus* and *M. mystacinus* . Fig. 4 shows this as a chain of Venn diagram relationships.

Note that the overlap (><) RCC-5 relationships between the taxonomic concepts of COL *M. levis*, *M. rossiaemeridionalis* and *M. arvalis* in MDD cannot be inferred from name-based alignments using string matching alone (c.f. Franz et al. (2016a)).

## Discussion

One of the major obstacles to widespread adoption of taxonomic concept alignments has been the difficulty of automating inference of RCC-5 relations to rapidly process thousands of checklist records. Name-based alignment tools are dominant in practice today because they are practical and simple. However, records in multiple checklists that match, based on their names, might refer to different taxonomic concepts in the checklists due to differences in name usage (circumscription) advanced by the parties preparing the checklists. A name from a record, therefore, does not refer to any single taxonomic concept outside the context of just a single checklist. We have presented a free and computationally practical algorithm to overcome this difficulty that is based on tracking how exemplar occurrences are differently assigned to accepted species across two taxonomies. Differing placements of occurrences permit accurate inference of taxonomic concept identity and other relationships.

While we do not provide a formal analysis of computational efficiency here, there are reasons to expect that List Tools will be efficient. There is no step in which every checklist A record is compared with every checklist B record. There is also no step involving general theorem proving or SAT-solving (c.f. Thau et al. (2008)). The method is, therefore, expected to scale in rough proportion to the number of records. The cost of the set operations is a concern (Redelings and Holder 2017), since the sets become as large as the checklists, but this is counterbalanced by the fact that there are few large sets and the total number of sets is limited by the height of the hierarchies (number of ranks). The difficulty of set operations over large trees does not seem to be an issue in practice and is borne out by the < 30 second run-time of the application above.

One limitation of our analysis is that we did not attempt to quantitatively validate the accuracy of the RCC-5 relations inferred by the List Tools algorithm. The algorithm we presented is grounded in objective set relationships, based on exemplars, but its accuracy is only as good as the quality and completeness of the exemplar set. When using type specimens as exemplars, the method is most effective when the checklists contain abundant synonymy information. For example, a split r → r’, s’ introducing a ‘new’ accepted record s’ with tc(r) > tc(s’), can only be detected if s’ has an exemplar not in r’. That requires that an occurrence known in tc(s’) is also known in tc(r), for example, in a synonym or subspecies of r. Concretely, if the *Peromyscus maniculatus* split example (above) did not have subspecies or synonyms in COL, i.e. if the names *P. sonoriensis*, *P. labecula* and *P. gambelii* were newly coined since COL had last updated its nomenclatural data, then we could not construct exemplars from these names because each exemplar requires an occurrence whose placement is known in both checklists. In consequence, we could not propose appropriate RCC-5 relationships between *P. maniculatus* according to COL and *P. sonoriensis*, *P. labecula* or *P. gambelii*. This data configuration (a demotion to synonym that later gets reversed or promotion of a subspecies) seems to be common in cases we’ve looked at. The utility of taxonomic alignments in general depends on the semantic precision and rigour that taxonomists bring to the definitions of taxa.

How far should the resulting alignment be trusted? One way to validate an alignment would be to check each relationship against expert opinion or against a knowledge base of taxonomic concepts that is comprehensive for the group in question. Preliminary experiments with the The Mammal Diversity Database show agreement between List Tools alignments of MDD versions pairs on the one hand and the MDD version difference reports published by MDD on the other. Regarding the validity of the present COL/MDD comparison, we are not aware of any good external knowledge base capable of doing it and expert review is not practical. Instead, the output is designed to enable triaging for the expert to review in a way that prioritises what matters for their application.

We therefore suggest that the broader value of the List Tools algorithm is in unlocking a positive feedback loop where automated inference of RCC-5 relations helps speed the manual review and validation steps needed for producing high-quality checklists and motivates the importance of nomenclatural metadata in taxonomic revisions. There are two main use cases we anticipate for this approach: aligning alternative taxonomies for the same group, such as those in the illustration we gave above between COL and MDD and aligning a new version of a taxonomy to an older version in order to document changes in taxonomic concepts, generate “diff” reports and flag potential issues for review. This latter application is especially valuable for large-scale taxonomies produced by collaborative efforts, such as monthly or annual versions of COL, but it can be useful to speed the process of generating RCC-5 relations even for single-author taxonomic monographs or revisions.

An important direction for future work is to incorporate the exemplar-based approach into the workflows of taxonomic authorities as they produce new checklist versions. ChecklistBank, for example, is a primary repository for the standard-compliant submission and retrieval of versioned biological classifications. It also provides an important tool for checklist authors and users to compare taxonomies as part of the validation process for data aggregation (e.g. in updates to the GBIF database) and taxonomy revision. List Tools provides the methods required for users to select pairs of checklists on ChecklistBank and efficiently generate alignments and associated reports from Darwin Core files.

Predictions from exemplar sets, when incorrect with respect to other evidence, could be brought into agreement with correct (intended) taxonomic concept relationships by strategically adding, removing or reassigning members of the exemplar set. For example, “moving” an exemplar from one record to another in one of the checklists can change a congruent = relationship into <, > or ><. This suggests additional curator-facing tools that could be added to processing pipelines and user workflows.

Another important ingredient for trustworthy alignment is the completeness and accuracy of the nomenclatural information in the two input checklists. This includes checking whether names from both lists are matched correctly and why some names remain unmatched across lists, for example, due to incomplete synonymy lists. As future work, one could also quantify the relative availability of information for alignment purposes. For example, one could calculate “exemplar density” as the number of exemplars available per species in a higher taxonomic unit. The number of unknown relationships (see Table 2) may also reflect potentially useful exemplars being missed due to nomenclatural mistakes or gaps.

The current software has a report generator that shows proposed taxonomic concept relationships, with exemplar set intersections and differences, for each pair of records for accepted species (where one record in each pair is in each checklist). Many other kinds of analyses and reports might be based on the same underlying ability to calculate RCC-5 relationships. Some reports that one might consider are:

- Summarising the information found in the complete species comparison report;
- Focusing on information expected to be of particular interest for curation, for example, excluding congruences (=) or highlighting overlaps (><);
- Summarising the differences between the checklists;
- Merging two checklists into a single synthetic checklist (Rees and Cranston 2017);
- Comparing taxonomic concepts at rank higher than species, for surfacing hierarchy differences;
- Comparing more than two checklists at once to create coordinated or unified reports;
- Reports tuned to the needs of particular workflows;
- Graphical illustrations of configurations of related taxonomic concepts;
- Presenting information on irregularities from the type specimen matching phase, such as ambiguous or doubtful matches.

Further resources supporting integration could include user tutorials, allowing new users to train on the tool and ultimately apply it towards their multi-taxonomy management foci. Building up a user base through ChecklistBank or specific taxonomic authorities can inform the next phase of providing reports for the most common alignment tasks. The List Tools alignment method can also be integrated into the validation and updating process of open source platforms, such as GitHub that some taxonomic authorities use for dissemination purposes. In general, we assume that: (1) further developing the technical capabilities of List Tools and (2) making the case for wider implementation through gradual user-driven involvement, are parallel developments that will need to stay closely connected.

The source code for the programme that implements the method and that was used to generate the example is available as a Zenodo repository (Rees 2026).

## Supplementary Material

619E03D1-E926-500E-BBB9-A4A30FE1FF09 10.3897/BDJ.14.e191754.suppl1 Supplementary material 1 Supplementary Table 1 RCC5 Report Data type Taxonomic alignment Brief description Table of RCC-5 relationships inferred using the List Tools method between the Mammal Diversity Database version 2.0 and Catalogue of Life version 2024 File: oo_1550790.csv https://binary.pensoft.net/file/1550790 Jonathan Rees

BA2C57B0-1FFD-5A1A-81F3-E64524DF82C8 10.3897/BDJ.14.e191754.suppl2 Supplementary material 2 Supplementary Table 2 Exemplars Report Data type Taxonomic alignment Brief description Table of exemplars (shared occurrences amongst checklists) derived from nomenclatural information in Mammal Diversity Database version 2.0 and Catalogue of Life version 2024 File: oo_1550791.csv https://binary.pensoft.net/file/1550791 Jonathan Rees

## Figures and Tables

**Table 1.** Logically consistent relationships between two taxonomic concepts, based on a given relationship between the exemplar sets of their associated records.

Relationship between exemplar sets es(r) and es(s)	Relationships between taxonomic concepts tc(r) and tc(s) that are consistent with this exemplar set relationship
=	=, <, >, ><
<	<, ><
>	>, ><
!	!, =, <, >, ><
><	><

**Table 2.** Summary of relationships between accepted species names inferred by List Tools for MDD 2.0 and COL 2024 checklists. The RCC-5 relationships listed (=,<,>,><) describe the number of estimated set relationships between taxonomic concepts in the two checklists, ignoring disjointness (!) relationships. “Unknown relationship” means a record has no exemplars and, therefore, the method has no way to determine a relationship with any particular species in the other checklist. Usually this just means there is no species with the same name in the other checklist and reflects differences in curation priorities, scientific knowledge at the time the checklists were prepared or of scientific or taxonomic opinion.

**Relationship**	**Count**
Is congruent with (=)	5175
Contains (>)	492
Is contained in (<)	340
Overlaps (><)	122
In MDD, unknown relationship to COL	880
In COL, unknown relationship to MDD	307

**Table 3.** An example congruence (=) RCC-5 relationship between the concepts for accepted names in COL and MDD checklists. The three right-hand columns list exemplars, based on whether they are found in both concepts associated with the accepted names or only one concept. Each exemplar is labelled by an assigned numerical ID and the stemmed epithet of its associated name.

**Proposed Relationships**			**Exemplars**		
**COL Name**	**RCC5**	**MDD Name**	**In both COL and MDD concepts**	**In COL concept, but not MDD concept**	**In MDD concept, but not COL concept**
Caluromys lanatus	=	Caluromys lanatus	1031 cicur; 1091 lanat; 1087 natterer; 1077 ochrop; 1059 ornat; 1035 uitalin	None	None

**Table 4.** Examples of “contains” (>) RCC-5 relationships between the accepted name “Peromyscus maniculatus” in COL and multiple accepted names in MDD. The three right-hand columns list exemplars, based on whether they are found in both concepts associated with the accepted names or only one concept. Each exemplar is labelled by an assigned numerical ID and the stemmed epithet of its associated name.

**Proposed Relationships**			**Exemplars**		
**COL Name**	**RCC5**	**MDD Name**	**In both COL and MDD concepts**	**In COL concept, but not MDD concept**	**In MDD concept, but not COL concept**
Peromyscus maniculatus	>	Peromyscus gambelii	10815 anacap; 2716 assimil; 10531 catalin; 10835 cinerit; 10781 clement; 10853 coolidge; 645 dorsal; 10839 dub; 10735 elus; 10757 exigu; 10845 exter; 10777 geronimens; 10717 huey; 1147 imperfect; 9887 magdalen; 10773 margarit; 5164 med; 10831 orester; 10847 sanctaeros; 10523 santacruz; 10283 streator	10857 abietor; 6979 alpin; 5820 angust; 10843 anticostiens; 6861 argentat; 10731 balaclau; 10723 bland; 7172 boreal; 10813 erem; 4496 fulu; 10817 georgiens; 10729 gunnison; 10827 hollister; 10733 inclar; 10719 labecul; 7021 lute; 10859 maniculat; 10725 nubiterr; 10849 ozarkiar; 7806 pallescens; 10793 perimekur; 10791 plumbe; 1679 rubid; 7668 saturat; 10035 saxamans; 10783 serrat	None
Peromyscus maniculatus	>	Peromyscus labecula	10723 bland; 4496 fulu; 10719 labecul	10857 abietor; 6979 alpin; 10815 anacap; 5820 angust; 10843 anticostiens; 6861 argentat; 2716 assimil; 10731 balaclau; 7172 boreal; 10531 catalin; 10835 cinerit; 10781 clement; 10853 coolidge; 645 dorsal; 10839 dub; 10735 elus; 10813 erem; 10757 exigu; 10845 exter; 10817 georgiens; 10777 geronimens; 10729 gunnison; 10827 hollister; 10717 huey; 1147 imperfect; 10733 inclar; 7021 lute; 9887 magdalen; 10859 maniculat; 10773 margarit; 5164 med; 10725 nubiterr; 10831 orester; 10849 ozarkiar; 7806 pallescens; 10793 perimekur; 10791 plumbe; 1679 rubid; 10847 sanctaeros; 10523 santacruz; 7668 saturat; 10035 saxamans; 10783 serrat; 10283 streator	None
Peromyscus maniculatus	>	Peromyscus sonoriensis	6979 alpin; 5820 angust; 10731 balaclau; 7172 boreal; 10817 georgiens; 10729 gunnison; 10827 hollister; 10733 inclar; 7021 lute; 10849 ozarkiar; 7806 pallescens; 10793 perimekur; 1679 rubid; 7668 saturat; 10035 saxamans; 10783 serrat	10857 abietor; 10815 anacap; 10843 anticostiens; 6861 argentat; 2716 assimil; 10723 bland; 10531 catalin; 10835 cinerit; 10781 clement; 10853 coolidge; 645 dorsal; 10839 dub; 10735 elus; 10813 erem; 10757 exigu; 10845 exter; 4496 fulu; 10777 geronimens; 10717 huey; 1147 imperfect; 10719 labecul; 9887 magdalen; 10859 maniculat; 10773 margarit; 5164 med; 10725 nubiterr; 10831 orester; 10791 plumbe; 10847 sanctaeros; 10523 santacruz; 10283 streator	None
Peromyscus maniculatus	>	Peromyscus maniculatus	10857 abietor; 10843 anticostiens; 6861 argentat; 10813 erem; 10859 maniculat; 10725 nubiterr; 10791 plumbe	6979 alpin; 10815 anacap; 5820 angust; 2716 assimil; 10731 balaclau; 10723 bland; 7172 boreal; 10531 catalin; 10835 cinerit; 10781 clement; 10853 coolidge; 645 dorsal; 10839 dub; 10735 elus; 10757 exigu; 10845 exter; 4496 fulu; 10817 georgiens; 10777 geronimens; 10729 gunnison; 10827 hollister; 10717 huey; 1147 imperfect; 10733 inclar; 10719 labecul; 7021 lute; 9887 magdalen; 10773 margarit; 5164 med; 10831 orester; 10849 ozarkiar; 7806 pallescens; 10793 perimekur; 1679 rubid; 10847 sanctaeros; 10523 santacruz; 7668 saturat; 10035 saxamans; 10783 serrat; 10283 streator	None

**Table 5.** Examples of “contains” (>) and “overlap” (><) RCC-5 relationships between the concepts associated with accepted names “*Microtus arvalis*” and “*Microtus levis*” in COL and four accepted names in MDD. The three right-hand columns list exemplars, based on whether they are found in both concepts associated with the accepted names or only one concept. Each exemplar is labelled by an assigned numerical ID and the stemmed epithet of its associated name.

**Proposed Relationships**			**Exemplars**		
**COL Name**	**RCC5**	**MDD Name**	**In both COL and MDD concepts**	**In COL concept, but not MDD concept**	**In MDD concept, but not COL concept**
Microtus arvalis	><	Microtus arvalis	5330 angular; 5416 arual; 5342 asturian; 5308 calyps; 5280 cimbric; 5384 hawelk; 5274 incognit; 5312 meridian; 5276 orcadens; 5318 ronaldshaiens; 5382 sandayens; 5362 sarn; 5390 terrestr; 5404 westr	5352 breuirostr; 5266 gudauric; 5364 hyrcan; 5380 iphigeni; 5408 khorkoutens; 5406 macrocran; 803 obscur; 5414 transcaucasic; 5282 transuralens	5242 leu
Microtus arvalis	>	Microtus mystacinus	5364 hyrcan; 5408 khorkoutens	5330 angular; 5416 arual; 5342 asturian; 5352 breuirostr; 5308 calyps; 5280 cimbric; 5266 gudauric; 5384 hawelk; 5274 incognit; 5380 iphigeni; 5406 macrocran; 5312 meridian; 803 obscur; 5276 orcadens; 5318 ronaldshaiens; 5382 sandayens; 5362 sarn; 5390 terrestr; 5414 transcaucasic; 5282 transuralens; 5404 westr	None
Microtus arvalis	>	Microtus obscurus	5352 breuirostr; 5266 gudauric; 5380 iphigeni; 5406 macrocran; 803 obscur; 5414 transcaucasic; 5282 transuralens	5330 angular; 5416 arual; 5342 asturian; 5308 calyps; 5280 cimbric; 5384 hawelk; 5364 hyrcan; 5274 incognit; 5408 khorkoutens; 5312 meridian; 5276 orcadens; 5318 ronaldshaiens; 5382 sandayens; 5362 sarn; 5390 terrestr; 5404 westr	None
Microtus levis	>	Microtus rossiaemeridionalis	5240 muhlis; 5232 relict; 5238 rossiaemeridional	5242 leu	None
